# Emerging Ocular Pathogen Resistance and Clinically Used Solutions: A Problem That Is More than Meets the Eye

**DOI:** 10.3390/ph19010031

**Published:** 2025-12-23

**Authors:** Marusha Ather, Christopher D. Conrady

**Affiliations:** 1 University of Nebraska Medical Center, Omaha, NE 68198, USA; 2 Department of Ophthalmology and Visual Sciences, John Moran Eye Center, University of Utah, Salt Lake City, UT 84132, USA

**Keywords:** eye, resistance, endophthalmitis, virus, infection, pathogen, multi drug resistant

## Abstract

**Background/Objectives:** Antimicrobial resistance (AMR) in ocular infections has become a serious concern with major implications for vision preservation. Bacterial AMR contributed to 4.71 million deaths worldwide in 2021, and ophthalmology mirrors these trends with multidrug resistance rates as high as 66% documented in some regions and persistently high methicillin resistance among common ocular pathogens. Across regions and care settings, traditional empiric therapies are losing effectiveness against an expanding range of pathogens, resulting in slower recovery, more complications, and, in many cases, permanent vision loss. This review aims to synthesize recent clinical, microbiologic, and pharmacologic evidence on ocular AMR, focusing on recent studies to capture current resistance patterns, therapeutic challenges, and evolving management strategies. **Methods:** Most included papers were published between 2020 and 2025, with additional foundational studies referenced where appropriate. Reports and systematic reviews addressing bacterial, viral, fungal, and parasitic ocular pathogens were evaluated to characterize current resistance mechanisms and management strategies across ocular pathogens. **Results:** The eye’s anatomic and physiologic barriers limit drug penetration, often promoting resistance and reducing therapeutic efficacy. Resistance mechanisms vary by pathogens; *Pseudomonas* keratitis is driven mainly by efflux pumps and biofilm formation, while CMV retinitis’ mutations in UL97 and UL54 are linked with clinical failure, and in MRSA associated *Staphylococcus* keratitis, the presence of mecA necessitates vancomycin-based therapy across bacterial, viral, fungal, and parasitic infections, with mechanisms such as β-lactamase production, efflux pump overexpression, target-site mutation, and biofilm formation contributing to poor response to standard therapy. MDR *Pseudomonas* keratitis remains the leading cause of rapidly progressive corneal infection with high risk of perforation and vision loss, while resistant CMV retinitis continues to threaten sight in immunocompromised patients despite antiviral advances. MDR organisms are recalcitrant to treatment and may lead to longer treatment courses and potentially worse outcomes and are discussed in detail within the manuscript. **Conclusions:** Ocular AMR represents an urgent and expanding clinical challenge. This review centers on the two most encountered multidrug-resistant organisms and their corresponding ocular sites, *Pseudomonas aeruginosa* (anterior segment) and CMV (posterior segment), while contextualizing them within the broader spectrum of resistant bacterial, viral, fungal, and parasitic pathogens. Despite growing awareness of AMR in ophthalmology, comprehensive surveillance data and longitudinal epidemiologic studies remain limited, making it difficult to track evolving resistance trends or guide region-specific therapy. Preserving vision in the AMR era will require faster diagnostics, improved ocular drug-delivery systems, and pathogen-specific therapies.

## 1. Introduction

### 1.1. The Emergence of Drug Resistance in Ophthalmology

Antimicrobial resistance (AMR) has become one of the most urgent global health threats, contributing to millions of deaths each year. In 2019, an estimated 13.7 million infection-related deaths occurred worldwide, with 7.7 million associated with 33 bacterial pathogens the authors studied, underscoring the magnitude of bacterial infections and their resistance potential across medical disciplines [1]. In 2021, 4.71 million deaths were estimated to be associated with bacterial AMR, with an 80% rise in adults over 70, with an associated USD 3.5 billion per year financial loss [2,3]. Ophthalmology mirrors these trends: drug-resistant ocular pathogens are increasingly reported and have become a critical cause of vision-threatening infections worldwide. The Antibiotic Resistance Monitoring in Ocular Microorganisms (ARMOR) surveillance program analyzed isolates collected between 2009 and 2018 and revealed persistently high methicillin resistance among approximately one-third of *Staphylococcus aureus* and nearly half of coagulase-negative staphylococci and roughly three-fourths of methicillin-resistant isolates displayed multidrug resistance, demonstrating that ocular pathogens are subject to resistance pressures comparable to those observed systemically [4]. While ARMOR data reflects U.S. surveillance, regional variation is substantial since prevalence rates of multidrug-resistant ophthalmic infections are estimated to be as high as 66.06% in Ethiopia, for example, highlighting differences in healthcare access and antimicrobial use patterns [4,5]. Clinically, resistant ocular infections are associated with delayed responses to empiric therapy, higher complication rates, and poorer visual outcomes, often requiring fortified or systemic antibiotics, surgical intervention, or prolonged hospitalization [4]. The growing clinical impact of AMR in ophthalmology was shown by the 2023 U.S. outbreak of extensively drug-resistant *Pseudomonas (P.) aeruginosa* (VIM-GES-CRPA) linked to contaminated artificial tears. This outbreak resulted in 81 infections across 18 states, leading to 14 cases of vision loss, 4 enucleations, and 4 deaths within 30 days of culture collection [6].

Although ocular infections represent a small fraction of all infectious diseases, the eye’s structural and immunologic isolation creates a unique clinical environment. Anatomically, it is protected by the tear film, corneal epithelium, and blood-ocular barriers, restricting microbial invasion but also limiting therapeutic drug penetration [7,8,9]. Consequently, ocular infections can progress rapidly, and delayed or suboptimal therapy often results in irreversible vision loss. Despite the severity, ocular pathogens remain underrepresented in global AMR surveillance networks, especially among low- and middle-income countries where laboratory and health information systems cannot adequately support population-level AMR monitoring [10,11]. Furthermore, surveillance efforts also predominantly emphasize bacterial pathogens despite fungal keratitis representing 37–72% of microbial keratitis in tropical and subtropical clinical series, while the UK reports only 0.32 cases per million annually [12]. This discrepancy crucially limits regional drug-resistance insight and evidence-based management guidelines.

### 1.2. Ocular Anatomy and Pathogen Susceptibility

The eye’s anatomy is a double-edged sword: its barriers preserve vision by blocking pathogens, yet those same defenses limit the effectiveness of many treatments. Unique anatomical barriers significantly impact ophthalmological drug delivery, with effectiveness depending on the route of administration [8,9,13].

For topical delivery, precorneal factors dominate drug loss and efficacy. Rapid tear turnover (~0.5–2.2 μL/min) and nasolacrimal drainage remove 80–95% of instilled drugs within minutes [8,13]. The cornea’s multilayered architecture, composed of alternating hydrophobic and hydrophilic domains, governs drug permeability [14,15,16]. Structurally, the cornea comprises five layers: epithelium, Bowman’s membrane, stroma, Descemet’s membrane, and endothelium, with the epithelium and endothelium serving as the principal diffusion obstacles [17]. The epithelium, formed by multiple layers of stratified squamous cells with tight junctions containing occludin, claudin, and zonula adherens proteins, acts as a lipophilic barrier that predominantly restricts paracellular passage of hydrophilic molecules, with permeability generally decreasing for compounds exceeding approximately 500 Da, though lipophilicity and molecular characteristics significantly influence actual penetration [13,18]. Beneath it, the stroma, which constitutes 90% of the corneal thickness, is highly hydrated and forms the major barrier for lipophilic compounds due to its collagen- and proteoglycan-rich matrix [8,19]. The endothelium, a monolayer of hexagonal cells, maintains the slightly dehydrated state for optical transparency of the cornea and permits the selective passage of small hydrophilic solutes through leaky tight junctions [14,15].

For systemic and posterior segment delivery, the blood–ocular barriers are critical and impose further difficulties in drug administration. The blood-aqueous barrier (formed by the non-pigmented ciliary epithelium and endothelial cells) and blood-retinal barrier (comprising the retinal pigment epithelium [RPE] and Bruch’s membrane of the outer barrier and retinal vascular endothelium, pericytes, and astrocytes of the inner barrier) restrict drug penetration through tight junctions containing claudins, occludins, and junctional adhesion molecules [13]. These barriers limit passage of large molecules and prevent macromolecule accumulation in intraocular tissues [13]. Efflux pumps such as P-glycoprotein actively expel drugs from ocular tissues, reducing the effectiveness of antibiotics and other therapies. In addition, metabolic enzymes in the corneal and conjunctival epithelium degrade many compounds before absorption, while conjunctival blood flow further enhances drug clearance [8].

Collectively, these barriers explain why topical drug bioavailability typically remains below 5% for most conventional formulations, often necessitates repeated dosing to maintain therapeutic levels, increasing the likelihood of corneal toxicity and patient noncompliance, and has been hypothesized to promote bacterial resistance [8,20]. Consequently, the development of alternative drug delivery platforms including nanomaterials, plasmid/gene therapies, and drug-eluting contact lenses is critical in combating emerging resistance and hard to treat pathogens within the eye [9].

### 1.3. Spectrum of Ocular Pathogens by Anatomical Site

Exogenous infections primarily affect anterior segment structures. Surface infections include blepharitis (eyelid margin inflammation commonly caused by *Staphylococci*, *Streptococcus pyogenes*, *P. aeruginosa*, and *Demodex* species), bacterial conjunctivitis (*Staphylococcus aureus*, *Streptococcus pneumoniae*, and *Haemophilus influenzae*), and infectious scleritis (often associated with *P. aeruginosa* in post-surgical cases) [21]. Contact lens-associated microbial keratitis (2–20 per 10,000 wearers annually) involves corneal epithelium and stroma, predominantly caused by *P. aeruginosa*, with *Staphylococcus*, *Acanthamoeba*, and fungi (*Fusarium*, *Candida*, *Aspergillus*) as additional pathogens that very rarely spread to posterior segment structures [22,23,24,25]. Penetrating ocular trauma introduces environmental organisms (*Coagulase negative Staphylococci*, *Bacillus*, and *P. aeruginosa*) into the anterior and vitreous chambers, with post-traumatic endophthalmitis occurring in 1–3% of injuries with especially high rates (7.5–13.5%) seen with retained intraocular foreign bodies [26,27,28]. Procedural inoculation introduces pathogens into sterile spaces: post-cataract endophthalmitis (0.012–0.08%) and post-intravitreal injection endophthalmitis (0.02–0.08% per injection) involve the anterior chamber and vitreous, with *coagulase-negative staphylococci* (66–70% post-cataract), *Streptococcus* species, and *Propionibacterium acnes* in delayed presentations but there are many other potential pathogens [29,30,31,32,33,34,35].

Endogenous infections (2–8% of endophthalmitis cases) seed posterior structures (choroid, retina, and vitreous) via hematogenous spread in immunocompromised or very sick hosts [36,37]. Bacterial endogenous endophthalmitis from *Staphylococcus aureus* bacteremia and *Klebsiella pneumoniae* liver abscesses can cause chorioretinitis and vitritis [38]. Fungal endophthalmitis from *Candida* species occur in 1.8–3.6% of fungemia cases when patients undergo ophthalmologic screening, while viral retinitis from Cytomegalovirus (CMV) retinitis occurs primarily in severely immunocompromised hosts [39,40,41]. Herpes simplex virus and varicella-zoster virus can cause acute retinal necrosis, which typically occurs in immunocompetent individuals, or progressive outer retinal necrosis, a more aggressive form seen almost exclusively in severely immunocompromised patients [42,43]. Invasion of ocular tissues with these pathogens can result in significant local tissue damage, potentially limiting vision potential, requiring empiric treatment in most cases due to the time needed for pathogen and susceptibility identification. Many of these organisms, including multidrug-resistant (MDR) *Staphylococcus* species, *Klebsiella*, *Candida*, *CMV*, and *Pseudomonas*, have been shown to affect the eye [4,44,45,46,47,48,49]. Due to their visual significance, higher rates of resistance, and the more commonly resistant pathogens being encountered in ocular disease, CMV and Pseudomonas will serve as the primary focus of anterior and posterior segment resistant diseases below.

## 2. Clinical Importance of Drug Resistance in Ophthalmology

### 2.1. Most Clinically Relevant Drug-Resistant Infections

Among sight-threatening ocular infections with documented AMR concerns, bacterial keratitis, particularly contact lens-associated *Pseudomonas* infections, and CMV retinitis in immunocompromised patients, represent major clinical challenges due to their documented resistance patterns and poor visual outcomes [50,51,52]. For clinical context, drug-resistant *Pseudomonas* is the most commonly identified cause of bacterial keratitis from resistant organisms and rates of CMV resistance are much higher than other herpes viruses such as herpes simplex virus type 1 (30.04% to 1.38%, respectively) [53,54,55].

### 2.2. Pseudomonas Keratitis

Epidemiologically, *P. aeruginosa* consistently ranks among the most prevalent Gram-negative pathogens in bacterial keratitis worldwide and is the dominant organism in contact lens-associated ulcers, often associated with worse outcomes [56]. Across large surveillance studies, it accounts for about 8% of all bacterial keratitis isolates and 25–60% of culture-positive ulcers in contact lens wearers, revealing its strong association with biofilm formation and microtrauma from lens wear [23,57]. Recent outbreaks have also identified *P. aeruginosa* as a major cause of keratitis among patients exposed to contaminated artificial tear products [58,59]. Clinically, the infection has an acute and aggressive onset, with patients often presenting within hours with severe ocular pain, redness, tearing, photophobia, and decreased vision, reflecting both epithelial and stromal involvement [60]. Multidrug-resistant (MDR) *P. aeruginosa* keratitis infections are especially aggressive, showing a higher likelihood of corneal melting within 48–96 h and corneal perforation within 2–5 days [50,51,61]. These cases often respond poorly to empiric therapy, with delayed epithelial healing, recurrent or bilateral disease, and worse visual outcomes typically requiring prompt surgical intervention, reflecting both the organism’s virulence and the growing limitation of available antibiotics [51].

Management of *P. aeruginosa* keratitis centers on rapid initiation of intensive topical therapy to halt corneal destruction. Since the infection can perforate the cornea within hours, treatment is empiric and guided by risk factors such as contact lens usage [62]. Current guidelines emphasize tailoring therapy to ulcer severity and patient risk profile while maintaining intensive dosing early on to prevent stromal melt and perforation [62]. The two main regimens include topical fluoroquinolone monotherapy and fortified antibiotic combinations. Fluoroquinolones achieve excellent corneal penetration and provide broad Gram-negative coverage, making them suitable for small or peripheral ulcers [63]. For larger and more severe ulcers or the presence of a hypopyon, ophthalmologists employ fortified aminoglycosides with cephalosporin, typically tobramycin or gentamicin, alternated with cefazolin, providing synergistic bactericidal activity for deeper stromal involvement [64,65,66]. Regional resistance patterns are crucial, as studies from Iran recommend empiric concurrent use of ceftazidime with amikacin or ciprofloxacin for contact lens ulcers, given that all *P. aeruginosa* isolates in that region were resistant to vancomycin and cefazolin [65]. This is in contrast to a large study from Asia that found the cumulative rate of resistance was highest to polymyxin B and ciprofloxacin [67]. Adjunctive corticosteroids given after 2–3 days of antibiotic therapy can improve long-term vision in bacterial keratitis, as shown in the SCUT trial for non-*Nocardia* ulcers, but the use of anti-inflammatories is outside the scope of this review and can be found elsewhere [62]. Additionally, in the treatment of *P. aeruginosa* ulcers, a subgroup analysis from the SCUT trial did not find any benefit to topical corticosteroids [68].

MDR *P. aeruginosa* is defined as resistance to ≥1 antibiotic in ≥3 classes where susceptibility is typically expected (penicillins, cephalosporins, fluoroquinolones, aminoglycosides, and carbapenems) and is the most frequently encountered MDR organism in bacterial keratitis [55,69]. “Difficult-to-treat” (DTR) *P. aeruginosa* represents isolates nonsusceptible to all first-line anti-pseudomonal β-lactams, including piperacillin–tazobactam, ceftazidime, cefepime, aztreonam, meropenem, imipenem–cilastatin, ciprofloxacin, and levofloxacin [69]. When keratitis caused by *P. aeruginosa* shows clinical deterioration or poor response to fluoroquinolone therapy, escalation is guided by assessment of the size of the epithelial defect, size and depth of the infiltrate, degree of pain, and anterior chamber reaction. Testing for MDR or DTR strains should be considered. For MDR infections, treatment options may include topical formulations of systemic antibiotics, such as polymyxin E (colistin 0.19%), or emerging agents such as cefiderocol, or the use of additional agents including imipenem, tobramycin, polymyxin B, and rifampin, may be warranted, but those often require compounding [70,71,72,73] (Table 1). Additionally, resistance to these agents is an evolving landscape requiring third- or fourth-line agents [50,59]. Innovative therapies such as Rose Bengal photodynamic antimicrobial therapy (RB-PDAT) generate reactive oxygen species through green-light activation, producing radicals that damage bacterial DNA and membranes [38]. In vitro testing demonstrated complete inhibition of all extensively drug-resistant *P. aeruginosa* isolates, and two patients treated with RB-PDAT as an adjuvant to antibiotic therapy showed improved clinical and visual outcomes, although clinical evidence remains extremely limited and does not yet support established efficacy [50]. Other emerging strategies, including bacteriophage therapy, show promise in refractory infections and have been used successfully for life-threatening MDR *P. aeruginosa* lung colonization [74,75,76,77]. Emerging therapies such as RB-PDAT and bacteriophage therapies are still in their early stages of development for ocular disease. Despite aggressive management with newer antimicrobials and adjunctive therapies, visual outcomes in MDR *P. aeruginosa* keratitis remain poor, emphasizing the critical importance of infection prevention, contact lens hygiene, and antimicrobial stewardship to prevent the emergence of resistant strains [50,59,78,79].

Outside the cornea, MDR *P. aeruginosa* causes some of the most destructive infections encountered in ophthalmology. Intraocular disease, whether postoperative or endogenous, often progresses from acute inflammation to irreversible destruction within days, with over one-third of eyes requiring evisceration and fewer than 15% retaining ambulatory vision [47,80]. Panophthalmitis develops in approximately one-third of cases, representing the most severe manifestation of the infection [80]. Vitreous analyses in MDR *P. aeruginosa* endophthalmitis have revealed markedly elevated levels of IL-6, IL-8, IL-1β, TNF-α, and IL-10, an inflammatory profile that correlates closely with disease severity and poor visual outcomes [80]. In rare instances where infection extends posteriorly into the subretinal or scleral spaces, it remains refractory to both medical and surgical intervention, often resulting in bilateral blindness and enucleation despite maximal therapy [47].

### 2.3. Drug-Resistant CMV

In contrast to the acute anterior segment destruction typical of *P. aeruginosa* keratitis, CMV retinitis is a chronic posterior segment infection characterized by areas of retinal edema and necrosis with associated hemorrhage and inflammatory changes surrounding retinal vessels [39,42,81]. The virus reaches the retina via hematogenous spread, where it targets retinal vascular endothelial cells and pericytes, damaging the inner blood-retinal barrier and leading to loss of vascular integrity [82]. This vascular injury underlies the characteristic necrotizing retinitis that can progress to scarring, retinal detachment, and irreversible vision loss [82]. Despite antiviral therapy, visual outcomes remain variable, with many eyes showing stable or worsening acuity and complications such as immune recovery uveitis or retinal detachments occurring [81].

Clinically, CMV retinitis was the most frequent ocular opportunistic infection and a leading cause of visual loss in patients with advanced AIDS before effective antiretroviral therapy (ART). With potent ART, the incidence declined by ≥90–95% in U.S. cohorts. Data from the Longitudinal Study of Ocular Complications of AIDS estimated 0.36 cases per 100 person-years in the ART era, with nearly all new cases occurring at CD4^+^ T cell counts below 50 cells/µL but with instances reported in patients with much higher counts [83]. However, CMV retinitis persists in patients with late HIV presentation or poor immune reconstitution and is associated with worse visual outcomes, including faster visual decline, greater retinal area progression, higher rates of second eye involvement, and increased mortality than those who achieve immune restoration (CD4^+^ T cell count >100 for three months) [84]. The disease also remains important in transplant recipients and patients in resource-limited regions with inadequate antiviral access [85]. Similarly to *P. aeruginosa*, CMV resistance is an emerging global threat especially in immunocompromised patients. In hematopoietic cell transplant recipients treated for first-time CMV infection, resistance can emerge during the first three weeks of therapy (10% for maribavir and 2.5% for valganciclovir) [86]. Among transplant recipients with suspected CMV who undergo genotypic testing, resistance mutations are detected in about 30% of samples [53]. Additionally, resistant CMV is associated with worse systemic outcomes, including mortality in those being treated for CMV retinitis [87,88].

All currently available anti-CMV drugs used for retinitis (ganciclovir/valganciclovir, foscarnet, and cidofovir) inhibit viral replication (virostatic), requiring prolonged therapy, particularly until immune recovery [89]. Since systemic anti-CMV drugs have limited vitreous penetration due to the blood-retinal barrier, and oral ganciclovir is further restricted by hepatic first-pass metabolism, intravitreal therapy is utilized for sight-threatening lesions and is often combined with systemic treatment to manage extraocular disease and reduce contralateral eye involvement [90,91]. Systemic ganciclovir or valganciclovir achieves only modest vitreous concentrations (0.9–1.2 mg/L), whereas intravitreal delivery produces markedly higher intraocular levels (>20 mg/L), providing better local control [90].

Resistance typically results from mutations in the UL97 kinase and/or UL54 DNA polymerase genes following prolonged exposure or subtherapeutic drug levels [92]. In AIDS cohorts receiving long-term ganciclovir, approximately 11% of patients developed resistant CMV within six months and up to 28% within nine months [93]. Among modern solid-organ transplant recipients, resistance occurs in 1–3% overall but in 10–18% of high-risk groups, such as donor-positive/recipient-negative or lung transplant patients [92]. Although most CMV retinitis cases respond to first-line agents, these data highlight that a clinically meaningful subset will fail therapy due to resistance.

Clinical failure of intravitreal cidofovir occurs in a small proportion of patients and may reflect selection of resistant CMV strains harboring UL97 or UL54 mutations after prior ganciclovir or cidofovir exposure [94]. Importantly, ocular CMV disease can result from localized infection with a resistant subpopulation, and resistance may arise independently within the eye, emphasizing the need to achieve adequate local drug concentrations and CMV resistance testing in refractory disease [94].

### 2.4. Established and New Drug Development for CMV Retinitis

Management of drug-resistant CMV retinitis often involves transitioning to second-line antivirals. Foscarnet and cidofovir remain the primary alternatives for ganciclovir-resistant infection (Table 1). Foscarnet is generally preferred, though its use is limited by significant nephrotoxicity and the need for careful hydration and frequent monitoring of creatinine levels. Cidofovir is similarly constrained by its cumulative renal toxicity [95]. Additionally, both intravenous and intravitreal cidofovir have been associated with anterior uveitis and prolonged ocular hypotony and have been largely replaced by other, safer alternatives [96]. Combination therapy of local and/or systemic ganciclovir and foscarnet has been used for a synergistic effect in refractory or difficult-to-treat cases [97]. More recently, the therapeutic landscape has expanded modestly with the approval of new antiviral agents. Maribavir, a *UL97* kinase inhibitor approved in 2021 for refractory or resistant CMV, has demonstrated efficacy in systemic transplant-related infections [98]. Although current guidelines discourage the use of maribavir for CMV retinitis due to poor intraocular penetration and reported treatment failures, isolated cases have described favorable outcomes when used as part of systemic combination therapy [89,98]. Another agent is letermovir, which was approved in November 2017 for CMV prophylaxis in CMV seropositive hematopoietic stem cell transplant recipients [99]. Letermovir acts on a distinct viral target—the terminase complex—but is approved solely for prophylactic use, and has only limited off-label use, in drug-resistant, active CMV retinitis, where it was noted to develop high rates of genotypically confirmed resistance [100]. With their poor intraocular and central nervous system penetration and high rates of acquired resistance, the use of letermovir and maribavir is likely very limited in CMV retinitis treatment, especially as monotherapy [101,102]. Beyond pharmacologic therapy, emerging CMV-specific T-cell therapy (adoptive immunotherapy) has shown potential benefit in persistent and multidrug-resistant infections, including in CMV retinitis [103,104,105,106]. With effective HIV therapy and vigilant prophylaxis in transplants, the overall incidence of CMV retinitis has declined dramatically in the U.S., becoming rare in HIV patients with access to care [83]. However, resistant CMV remains an important cause of morbidity in transplant recipients, hematopoietic stem cell recipients, and other immunosuppressed patients, where MDR infections often require multi-agent management approaches, of which many are unfortunately off-label or investigational in ocular disease [92].

### 2.5. Other Emerging Resistant Pathogens

Among ocular isolates, *Staphylococcus aureus* and coagulase-negative staphylococci (CoNS) remain the organisms most strongly associated with multidrug resistance. Data from the ARMOR surveillance program show persistently high but largely stabilizing methicillin-resistance rates- roughly one-third of *S. aureus* and nearly half of CoNS isolates-indicating that resistance pressures have plateaued rather than continued to rise. These methicillin-resistant strains frequently display cross-resistance to macrolides and fluoroquinolones, limiting the efficacy of empiric topical agents such as moxifloxacin and azithromycin. Clinical series corroborate that methicillin-resistant staphylococci remain the leading cause of MDR ocular infections, particularly in keratitis and postoperative endophthalmitis, and often require vancomycin as definitive therapy [11]. Emerging concerns about vancomycin susceptibility patterns further underscore the narrowing therapeutic margin against these pathogens [11]. Pediatric ARMOR data similarly demonstrate stable resistance trends, with high but non-increasing rates of staphylococcal and pneumococcal resistance and continued susceptibility among *Haemophilus influenzae* and *P. aeruginosa* isolates [107]. Collectively, these findings suggest that in recent years, MDR staphylococci continue to pose the greatest clinical challenge in ocular infections.

Fungal keratitis is becoming an increasingly important cause of vision loss worldwide, especially in tropical regions and among contact lens wearers [108]. *Fusarium* species, most often those within the *Fusarium solani* complex, remain the leading cause of infection, followed by *Aspergillus flavus* and *A. fumigatus* in many centers [109,110]. Treatment of *Fusarium* keratitis is particularly difficult due to limited antifungal susceptibility and voriconazole activity is variable among *Fusarium* species [110]. In contrast, *Aspergillus* infections tend to respond better to voriconazole, although natamycin remains the most reliable first-line agent for *Fusarium* ulcers [109,111,112]. Large susceptibility studies consistently show that natamycin offers the broadest in vitro coverage for *Fusarium*, while voriconazole is used for non-*Fusarium* molds [108,110]. Nonetheless, reports of *Fusarium* isolates exhibiting high minimum inhibitory concentrations to both natamycin and voriconazole signal emerging resistance that could limit future treatment options [109,110]. *Aspergillus* keratitis often invades deeply into the corneal stroma and is associated with a slower response to therapy and poor outcomes, frequently requiring surgical intervention [108,113]. Finally, the yeast *Candida auris* has recently been reported as a cause of severe endogenous endophthalmitis and panophthalmitis, notable for its extensive multidrug resistance and high mortality risk [114]. Additionally, other Candida species are of concern in endogenous endophthalmitis due to emerging resistance [45,115,116].

Acanthamoeba keratitis (AK) has shown a global resurgence, particularly among contact lens users. National surveillance studies in Europe, the United States, and Australia report a several-fold rise in incidence since the early 2000s [117,118]. The infection typically presents with severe ocular pain, epithelial defects, and, in advanced cases, stromal infiltrates, often masquerading as bacterial or herpetic keratitis and leading to delayed diagnosis [118,119]. Despite combination therapy with biguanides (polyhexamethylene biguanide, chlorhexidine) and diamidines (propamidine), many cases remain challenging to treat due to the inherent resistance of Acanthamoeba cysts to antimicrobial agents, with treatment failure occurring in approximately 39% of cases [117,118].

Ocular toxoplasmosis, caused by *Toxoplasma gondii*, remains the leading cause of infectious posterior uveitis worldwide [120]. Although pyrimethamine–sulfadiazine therapy remains the standard first-line regimen, current treatments do not eradicate the parasite or prevent recurrence [121]. Clinically, the disease manifests as focal necrotizing retinochoroiditis with adjacent pigmented scars in those with appropriate risk factors and following prior exposure, and recurrence rates of 60–86% have been reported depending on follow-up [121,122,123]. Variability in clinical response to commonly utilized antibiotics to treat toxoplasmosis has been attributed to drug resistance and strain cystogenesis capacity in birds but it is a controversial mechanism for treatment failure clinically [124,125,126].

Microsporidial keratitis is increasingly recognized worldwide, affecting both immunocompetent and immunocompromised hosts, particularly in tropical regions [119]. The superficial epithelial form often presents as keratoconjunctivitis that may resolve spontaneously or with topical therapy, whereas the less common but more severe stromal form carries a poor prognosis and tends to be resistant to medical therapy, with most cases requiring surgical excision [127].

## 3. Types of Drug Resistance and Mechanisms by Organism

### 3.1. Bacterial Resistance Mechanisms

#### 3.1.1. *Pseudomonas aeruginosa*

**Figure 1:**
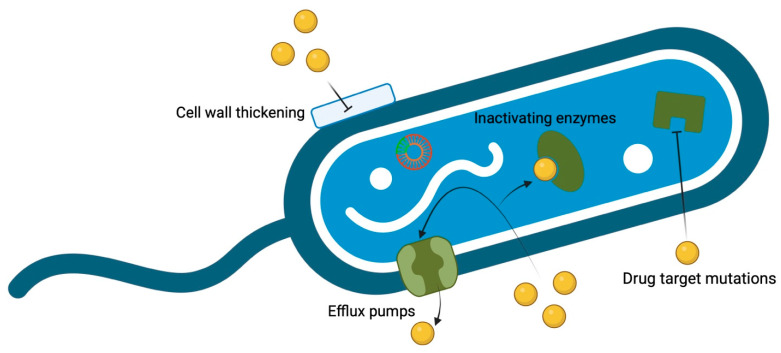
Mechanisms of bacterial resistance. Created in BioRender. Conrady, C. (2025) https://BioRender.com/j44k73z.

β-lactamase production represents a key resistance mechanism in *P. aeruginosa*, with ocular isolates frequently expressing chromosomal AmpC β-lactamase and acquired carbapenemases such as VIM, IMP, and GES-type enzymes, which hydrolyze most β-lactam antibiotics [128] (Figure 1). Recent outbreaks have identified clinical isolates carrying both VIM metallo-β-lactamase and GES-type ESBL, rendering them resistant to nearly all β-lactams [50]. Efflux pump overexpression, particularly MexAB-OprM and MexXY-OprM systems, actively expels multiple antibiotic classes including β-lactams, fluoroquinolones, and aminoglycosides [129]. Target site mutations in DNA gyrase (gyrA) and topoisomerase IV (parC) genes, particularly Thr83Ile and Ser87Leu substitutions, reduce fluoroquinolone binding affinity and are associated with frequent therapy failures involving ciprofloxacin or levofloxacin [130]. Biofilm formation is nearly universal among *P. aeruginosa* ocular isolates (with one study detecting it in 100% of keratitis and endophthalmitis cases), with biofilm-specific resistance genes such as *ndvB* and *tssC1* identified in 96.7% and 90.2% of isolates, respectively [131] (Figure 1). Biofilm formation is particularly relevant in contact lens-related infections, necessitating novel therapeutic approaches for effective treatment [132]. Type III secretion system genotypes show associations with distinct resistance patterns. In vitro studies have demonstrated that cytotoxic strains (exoU+) exhibit increased tolerance to contact lens disinfectants while clinical observational data suggest a trend toward higher fluoroquinolone MICs in invasive strains (exoS+), although this association has not consistently reached statistical significance [133,134]. Novel resistance gene combinations, such as *blaVIM-80* and *blaGES-9*, have been identified in extensively drug-resistant isolates that remain susceptible to cefiderocol, though other agents, including colistin and selected β-lactam/β-lactamase inhibitor combinations, can remain active depending on the strain [59,78,135]. In addition, *P. aeruginosa* often acquires resistance genes through mobile genetic elements such as integrons, transposons, and integrative conjugative elements, which enable horizontal gene transfer and the rapid spread of resistance among ocular isolates [136].

#### 3.1.2. Staphylococcal Resistance

Methicillin resistance in *Staphylococcus* species arises from the *mecA* gene, which encodes PBP2a, an altered penicillin-binding protein with reduced β-lactam affinity. This modification allows the pathogen to continue peptidoglycan synthesis even in the presence of β-lactam antibiotics [137]. This mechanism allows broad resistance to nearly all β-lactams except newer anti-MRSA agents, necessitating alternative non-β-lactam therapy such as vancomycin for ocular MRSA infections [138]. Vancomycin resistance remains rare among ocular isolates but involves two distinct mechanisms when present. Vancomycin-intermediate *S. aureus* (VISA) develops through cell wall thickening, which sequesters vancomycin within the outer peptidoglycan layers. In contrast, vancomycin-resistant *S. aureus* (VRSA) acquires *vanA* genes from enterococci, leading to the synthesis of D-alanyl-D-lactate peptidoglycan precursors that reduce vancomycin binding [139]. Only one case of VRSA keratitis has been documented from Eastern Europe to date, occurring as a recurrent corneal infection following penetrating keratoplasty [140]. Macrolide resistance in MRSA is typically mediated by *erm* genes encoding rRNA methyltransferases, *msr* genes encoding efflux pumps, and *mph* genes encoding phosphotransferases. Similar mechanisms are thought to occur in coagulase-negative staphylococci, although ocular-specific molecular data remain limited [141]. Clinically, surveillance studies reveal high erythromycin resistance rates—71.1% in pediatric ocular MRSA infections—and widespread azithromycin resistance, reported in up to 92.2% of methicillin-resistant isolates [142]. Fluoroquinolone resistance is also prevalent, ranging from 68% to 71% in MRSA within a large U.S. prospective study, while vancomycin resistance has not yet been broadly identified in ocular strains [49].

### 3.2. Mechanisms of Viral Resistance

#### 3.2.1. CMV Resistance

**Figure 2:**
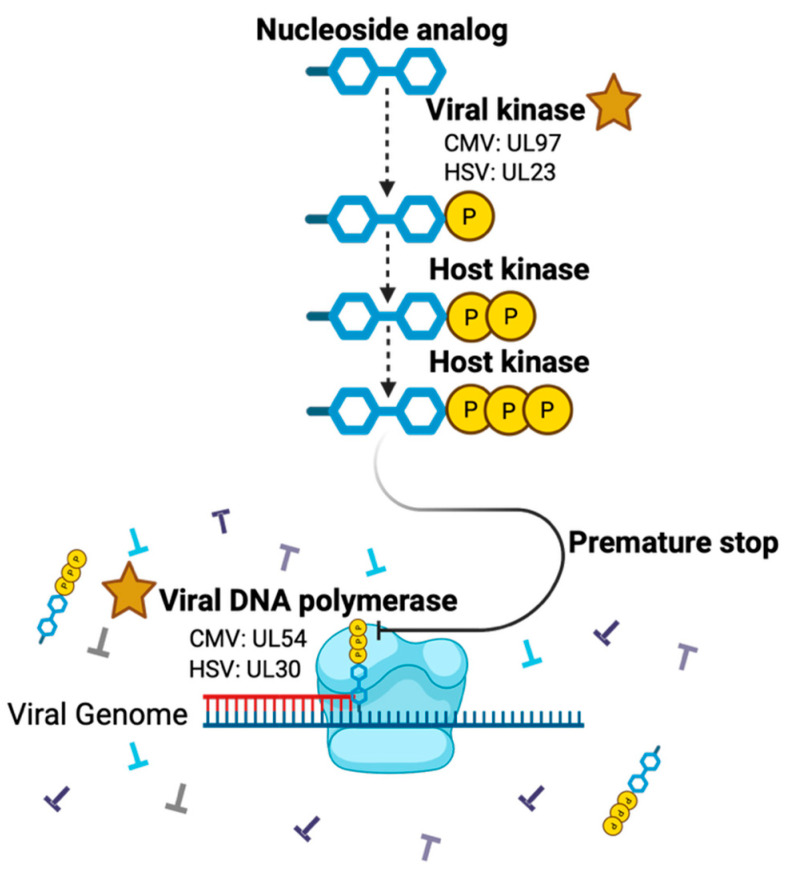
Mechanisms of herpesvirus nucleotide analog resistance. Created in BioRender. Conrady, C. (2025) https://BioRender.com/ifnzlo1. Stars—most common sites of resistance development.

Antiviral resistance in CMV retinitis mirrors systemic patterns and is driven primarily by mutations in UL97 (viral kinase) and UL54 (DNA polymerase) (Figure 2) [143]. UL97 substitutions prevent ganciclovir phosphorylation, producing high-level drug resistance while maintaining susceptibility to foscarnet and cidofovir [144]. Alterations in UL54 DNA polymerase can confer high levels of ganciclovir resistance and some cross-resistance to cidofovir and foscarnet [143]. When both UL97-UL54 mutations occur, resistance extends across multiple antivirals, often necessitating a switch to alternative agents such as foscarnet [145]. Baseline resistance is rare, with UL97 mutations found in roughly 0.5% of untreated cases, but prolonged ganciclovir exposure drives rapid selection, emerging in up to one-third of AIDS patients after three months of therapy [146,147]. CMV retinitis can develop resistance through mutations in the viral terminase complex, with mutations conferring letermovir resistance emerging rapidly in retinitis patients [100]. Mutations in *UL51* can amplify resistance three- to sevenfold when combined with *UL56* alterations, while *UL89* mutations confer cross-resistance to terminase inhibitors [148].

#### 3.2.2. HSV Resistance

In immunocompromised patients with treatment-refractory HSV keratitis, one of the leading causes of blindness related to infectious diseases, genotypic analyses show that approximately 83% of HSV-1-positive ocular samples harbor acyclovir-resistant strains due to mutations in *UL23* or *UL30* [149,150]. To restate, HSV-1 resistance is very low in the general population. The primary resistance mechanism involves mutations in the *UL23* gene encoding thymidine kinase, which account for most acyclovir-resistant cases, though mutations in the *UL30* gene encoding DNA polymerase can also cause drug resistance both to nucleoside analogs and foscarnet [151]. These TK-deficient mutants exhibit cross-resistance to all nucleoside analogs requiring viral phosphorylation, such as acyclovir, penciclovir, and ganciclovir, since these guanosine analogs depend on TK-mediated activation [152]. However, TK-deficient mutants remain susceptible to foscarnet, which inhibits the viral DNA polymerase directly. For resistant cases, current topical alternatives include trifluridine, foscarnet, and cidofovir [152]. However, it should be emphasized, though, that in the immunocompetent population the prevalence of acyclovir resistance is under 1% [153].

### 3.3. Fungal Resistance Mechanisms

Fungal ocular infections, including fungal keratitis and endophthalmitis, are most commonly caused by *Candida*, *Aspergillus*, and *Fusarium* species [154]. Fungal ocular infections present resistance challenges that extend beyond traditional molecular mechanisms. Traditional molecular mechanisms remain important, including azole resistance through *ERG11* or *cyp51A* mutations and efflux pump overexpression, amphotericin B resistance via alterations in ergosterol biosynthesis, and echinocandin resistance due to *FKS1* or *FKS2* mutations [155,156]. However, clinical resistance in ocular infections is often dominated by biofilm formation, resulting in limited drug penetration. Keratitis-associated fungi form dense, three-dimensional biofilms with interwoven hyphae that exhibit progressive, time-dependent resistance to all major antifungal classes [157]. Polymicrobial biofilms further amplify resistance, showing several-fold increased antimicrobial tolerance compared to single-species biofilms [158]. Mutations in the *Fusarium* CYP51 gene and efflux pumps provide broad species-level resistance to azoles [159,160].

Emerging melanized fungi such as *Curvularia lunata* and *Lasiodiplodia theobromae* display distinctive resistance patterns to amphotericin B, one of the main intravitreal antifungals, and multidrug-resistant isolates are associated with significantly poorer visual outcomes [161]. These combined resistance mechanisms contribute to treatment failures globally that necessitate approximately 100,000 enucleations or eviscerations worldwide annually due to fungal keratitis, highlighting the critical need for therapeutic strategies targeting biofilm disruption and enhanced drug delivery systems including combination therapy [162]. Additionally, an increasing MIC of isolated fungi from corneal scrapings is associated with increased odds of perforation [163]. Fungal MICs can help guide antifungal choice and we, and others, advocate for susceptibility testing when possible from corneal cultures [112,163,164,165].

### 3.4. Parasitic Resistance

Resistance in *Acanthamoeba* keratitis primarily stems from the organism’s ability to form double-walled cellulose cysts, which create an impermeable barrier to most antimicrobial agents [166,167]. Cyst wall synthesis can occur within hours of stress exposure, allowing rapid transition to a drug-tolerant state [168]. Additionally, efflux pumps such as *adeF*, *amrA*, and *amrB* expel antimicrobial agents and further decrease intracellular drug concentrations [169,170]. Biofilm formation on corneal surfaces and contact lenses further enhances survival and promote resistance [171]. These intrinsic structural features (double-walled cysts, efflux pumps, and biofilm formation) reduce antimicrobial efficacy.

### 3.5. Toxoplasma Gondii

Emerging evidence indicates that Toxoplasma gondii has developed multidrug resistance through diverse molecular pathways, although mechanisms differ between in vitro and human ocular isolates in humans. Antifolate resistance stems from mutations in the dihydrofolate reductase–thymidylate synthase complex, which reduce pyrimethamine binding and drug potency [99]. Additional substitutions in the cytochrome bc1 complex confer atovaquone resistance, while alterations in the apicoplast rRNA genes underlie macrolide resistance to clindamycin and azithromycin [126]. These genetic adaptations highlight the parasite’s ability to evade standard therapy, complicating long-term management of refractory ocular toxoplasmosis [126]. Evidence of drug resistance in the ocular toxoplasmosis is currently limited to a single isolate, TgCTBr11, obtained from newborns with congenital ocular toxoplasmosis. This isolate exhibited clear sulfadiazine resistance in experimental models but did not harbor the DHPS 407 mutation previously linked to sulfonamide resistance, indicating that resistant ocular strains may rely on alternative or currently unidentified mechanisms [126].

### 3.6. Microsporidia

Ocular microsporidiosis represents a challenging parasitic infection marked by highly specialized strategies of corneal invasion and long-term survival. After contact with contaminated water or soil, microsporidia use a polar filament apparatus to inject the infective sporoplasm directly into corneal epithelial cells or keratocytes, where they multiply within parasitophorus vacuoles inside the host cell [172]. Their thick chitin-rich spore wall and strictly intracellular lifestyle make them remarkably resistant to standard antimicrobial treatments, allowing the infection to persist even after therapy [127]. In some cases, these spores are hypothesized to remain dormant within the corneal stroma, leading to chronic infection or recurrence. The resulting inflammation can disrupt the eye’s natural immune balance and barrier function, similar to what is seen in other parasitic infections, ultimately contributing to corneal damage and vision loss [121,126,173,174,175].

## 4. Future Directions/Conclusions

Antimicrobial resistance in ocular infections has become a global concern with far-reaching implications for vision preservation, as seen through epidemiological data cited in the Introduction. Although resistance patterns vary across regions and healthcare settings, the overall trend is evident; traditional empiric therapies are losing effectiveness against an expanding range of pathogens. *P. aeruginosa* and methicillin-resistant *Staphylococcus* species continue to pose the greatest bacterial challenges, while growing resistance among viral pathogens, particularly *CMV* in immunocompromised patients, along with fungal organisms such as *Fusarium* and emerging and select parasites including *Acanthamoeba* and *Toxoplasma gondii*, reflects the widening scope of this problem. These infections often progress quickly, respond poorly to standard antimicrobial agents, and require surgical intervention, emphasizing the high stakes of delayed or ineffective treatment.

Progress in managing ocular antimicrobial resistance will require improvements in several areas. Rapid diagnostics, including molecular and sequencing-based methods, are needed to enable earlier pathogen identification, although their use is still limited by turnaround time, cost, and availability, and they may aid in pathogen-specific management and the improvement of outcomes [34,132,176,177,178]. More effective drug delivery systems are also needed, as traditional therapies deliver less than 5% of the dose into intraocular tissues [8,9,20,179]. A better understanding of pathogen mechanisms is also necessary, since much of the current evidence is based on in vitro, animal, or systemic non-ocular studies rather than clinical ocular isolates. Improvements in ocular pharmacokinetics and alternative delivery routes may further optimize intraocular drug exposure while reducing systemic toxicity [9,20,179]. Finally, expanding the regional and international surveillance of ophthalmic isolates is critical for tracking resistance trends and guiding empiric therapy and stewardship practices [176,177].

## Figures and Tables

**Table 1:** Drugs utilized in combating ocular infections.

	Drug	Topical	Subconjunctival	Intravitreal
**Antibacterial**	Azithromycin	10 mg/mL	-	-
	Amikacin	1.5–40 mg/mL	25–30 mg/0.5 mL	0.4 mg/0.1 mL
	Bacitracin	10,000 IU	-	-
	Ceftazidime	50 mg/mL	100 mg/0.5 mL	2.25 mg/0.1 mL
	Cefazolin	50 mg/mL	-	-
	Ceftriaxone	50 mg/mL	125 mg/0.5 mL	2 mg/0.1 mL
	Cefuroxime	5%	50 mg/0.5 mL	1 mg/0.1 mL
	Ciprofloxacin	0.30%	-	-
	Clarithromycin	10 mg/mL	-	-
	Colistin	0.19%	-	-
	Gentamicin	14 mg/mL	-	-
	Imipenem-cilastatin	1%	-	-
	Linezolid	2 mg/mL	-	-
	Moxifloxacin	0.50%	-	500 µg/0.1 mL
	Piperacillin/tazobactam	10%	-	250 µg/0.1 mL
	Tobramycin	14 mg/mL	-	-
	Vancomycin	15–50 mg/mL	25 mg/0.25 mL	1 mg/0.1 mL
**Antiviral**	Ganciclovir	0.15%	-	2–4 mg/0.05 mL
	Foscarnet	2.40%	-	1.2–2.4 mg/0.1 mL **
	Fomivirsen	-	-	330 µg/0.05 mL
	Acyclovir	3%	-	-
**Antifungal**	Caspofungin	0.50%	-	100 µg/0.1 mL *
	Amphotericin B	1.5 mg/mL	-	5–10 µg/0.1 mL
	Voriconazole	10 mg/mL	-	50–100 µg/0.1 mL
**Anti-parasitic**	Clindamycin	50 mg/mL	-	1 mg/0.1 mL

Drugs that have been utilized clinically but dosing, efficacy, and toxicity may not be completely understood. * limited data; ** no longer available.

## Data Availability

No new data were created or analyzed in this study. Data sharing is not applicable to this article.
